# Primary Sjögren’s Syndrome of Early and Late Onset: Distinct Clinical Phenotypes and Lymphoma Development

**DOI:** 10.3389/fimmu.2020.594096

**Published:** 2020-10-19

**Authors:** Andreas V. Goules, Ourania D. Argyropoulou, Vasileios C. Pezoulas, Loukas Chatzis, Elena Critselis, Saviana Gandolfo, Francesco Ferro, Marco Binutti, Valentina Donati, Sara Zandonella Callegher, Aliki Venetsanopoulou, Evangelia Zampeli, Maria Mavrommati, Paraskevi V. Voulgari, Themis Exarchos, Clio P. Mavragani, Chiara Baldini, Fotini N. Skopouli, Dimitrios I. Fotiadis, Salvatore De Vita, Haralampos M. Moutsopoulos, Athanasios G. Tzioufas

**Affiliations:** ^1^Department of Pathophysiology, School of Medicine, National and Kapodistrian University of Athens, Athens, Greece; ^2^Joint Rheumatology Academic Program, School of Medicine, National and Kapodistrian University of Athens, Athens, Greece; ^3^Unit of Medical Technology and Intelligent Information Systems, University of Ioannina, Ioannina, Greece; ^4^Proteomics Facility, Center for Systems Biology, Biomedical Research Foundation of the Academy of Athens, Athens, Greece; ^5^Department of Nutrition and Clinical Dietetics, Harokopio University of Athens, Athens, Greece; ^6^Rheumatology Clinic, Department of Medical Area, University of Udine, Udine, Italy; ^7^Rheumatology Unit, Department of Clinical and Experimental Medicine, University of Pisa, Pisa, Italy; ^8^Institute for Autoimmune Systemic and Neurological Diseases, Athens, Greece; ^9^Rheumatology Clinic, Department of Internal Medicine, Medical School, University of Ioannina, Ioannina, Greece; ^10^Department of Informatics, Ionian University, Corfu, Greece; ^11^Department of Physiology, Medical School, National and Kapodistrian University of Athens, Athens, Greece; ^12^Department of Biomedical Research, Institute of Molecular Biology and Biotechnology, Foundation for Research and Technology - Hellas, Ioannina, Greece; ^13^Chair Medical Sciences/Immunology, Academy of Athens, Athens, Greece

**Keywords:** primary Sjögren’s syndrome, data driven analysis, age group, lymphoma, clinical phenotype characteristics

## Abstract

**Objectives:**

To study the clinical, serological and histologic features of primary Sjögren’s syndrome (pSS) patients with early (young ≤35 years) or late (old ≥65 years) onset and to explore the differential effect on lymphoma development.

**Methods:**

From a multicentre study population of 1997 consecutive pSS patients, those with early or late disease onset, were matched and compared with pSS control patients of middle age onset. Data driven analysis was applied to identify the independent variables associated with lymphoma in both age groups.

**Results:**

Young pSS patients (19%, n = 379) had higher frequency of salivary gland enlargement (SGE, lymphadenopathy, Raynaud’s phenomenon, autoantibodies, C4 hypocomplementemia, hypergammaglobulinemia, leukopenia, and lymphoma (10.3% vs. 5.7%, p = 0.030, OR = 1.91, 95% CI: 1.11–3.27), while old pSS patients (15%, n = 293) had more frequently dry mouth, interstitial lung disease, and lymphoma (6.8% vs. 2.1%, p = 0.011, OR = 3.40, 95% CI: 1.34–8.17) compared to their middle-aged pSS controls, respectively. In young pSS patients, cryoglobulinemia, C4 hypocomplementemia, lymphadenopathy, and SGE were identified as independent lymphoma associated factors, as opposed to old pSS patients in whom SGE, C4 hypocomplementemia and male gender were the independent lymphoma associated factors. Early onset pSS patients displayed two incidence peaks of lymphoma within 3 years of onset and after 10 years, while in late onset pSS patients, lymphoma occurred within the first 6 years.

**Conclusion:**

Patients with early and late disease onset constitute a significant proportion of pSS population with distinct clinical phenotypes. They possess a higher prevalence of lymphoma, with different predisposing factors and lymphoma distribution across time.

## Introduction

The clinical phenotype of primary Sjögren’s syndrome (pSS) varies from a benign glandular disease to an aggressive systemic disorder, leading to end stage organ failure and lymphoma (1, 2). The extent and severity of pSS depends on many factors including individuals’ genetic background, environmental factors and several demographic features such as gender or age at onset. pSS affects primarily middle-aged females, although other age groups may also be involved. The functional status of the immune system is changing with age and it is reasonable to expect that such alterations may interfere with the clinical expression of the disease across different age groups. Apart from the immune system, other age-related parameters could potentially affect disease manifestations, such as hormones, prevalence of infections, various comorbidities, and medications. Therefore, differences in the disease phenotype across distinct age groups may reflect discrete underlying pathogenetic mechanisms.

Previous studies regarding the phenotype of pSS patients with early disease onset have shown that this subset is characterized by the presence of autoantibodies, lymphadenopathy, hypergammaglobulinemia, purpura, Raynaud’s phenomenon, and arthritis as compared to older patients (3–8). Very few groups have focused on pSS patients with late disease onset, describing only lower frequencies of various autoantibodies such as rheumatoid factor (RF), ANA, anti-Ro/SSA, or anti-La/SSB in comparison to younger pSS patients (4–6, 9, 10). However, the majority of these studies included a rather small number of patients with either early or late disease onset who were compared to the rest of the study population, as defined by the age cut off limits of each study. Interestingly, the association of age at pSS onset with lymphoma was reported only in one study (7). In the current work, a case-control study of pSS patients with early or late disease onset, matched with pSS control patients and disease onset at the typical 4^th^ or 5^th^ decade of life, is presented for the first time in the literature. Our aim was to investigate differences in the clinical picture between different pSS age groups and to explore the differential effect of age on lymphoma development by analyzing the independent lymphoma associated factors and the distribution pattern of lymphoma occurrence across pSS course.

## Patients and Methods

### Study Design

This is a retrospective, matched case-control study of a multicentre population of consecutive pSS patients, all fulfilling the 2016 ACR/EULAR classification criteria (11), who were followed up between May 1984 and May 2019, in 5 highly specialized pSS Rheumatology Centers from Greece and Italy (Universities of Athens, Pisa, Udine, Harokopio, and Ioannina) (UPAHI group). The study was approved by the local ethical committees of all the involved Institutions after obtaining patients’ informed consent and in compliance to general data protection regulations (GDPR). Cumulative clinical, laboratory, and histologic data were collected from medical charts until the last follow up of patients and were integrated in a final dataset, following a common reference model for Sjögren’s syndrome (12). Objective eye and oral tests, laboratory tests and minor salivary gland (MSG) biopsies were performed as the standard of care, according to physicians’ judgement usually at the time of diagnosis. Two study groups were identified: patients with pSS onset at ≤35 (young study group) and ≥65 years (old study group) of age. Every patient of either study group was matched with a pSS control patient according to gender and disease duration, whose age of pSS onset was within the 4^th^ or 5^th^ decade. pSS onset was defined as the year when the patient recalled disease-related manifestations, such as Raynaud’s phenomenon, arthritis, sicca symptoms, salivary gland enlargement (SGE), or purpura. Disease duration was calculated using as initial time point both the date of pSS onset and pSS diagnosis, and the time span between pSS onset and pSS diagnosis was also estimated. Every comparison among groups was performed based on clinical, laboratory/serological, and histologic features. Systemic organ involvement was defined as previously described by the ESSDAI domains (13). Fatigue was considered as present, if lasting more than three months between consecutive visits as recorded in the medical charts. The unified Greek and Italian young and old pSS patients were compared with their matched pSS middle aged control groups, respectively, and subsequently a comparison between Greek and Italian young and old pSS patients was performed to avoid source population bias. To explore the distribution pattern of lymphoma occurrence across the time course of pSS in the 2 age groups, we matched in 1:1 ratio the maximum possible number of early and late pSS lymphoma patients with middle aged pSS lymphoma controls according to pSS disease duration.

### Data Curation, Statistical Analysis and Data Driven Approaches

Automated medical data curation, presented in a previous study (14), was applied on the final dataset to deal with outliers, missing values and incompatible fields, as well as duplicated features. All outliers and incompatible values were finally removed from further analysis along with features having more than 50% missing values. Statistical analysis for categorical data was performed by chi-square test with Yates correction or Fisher exact when cell counts <5 patients and for numerical data with the Mann-Whitney or t-test, after Shapiro-Wilk normality test. The Fast-Correlation based feature selection (FCBF) algorithm was applied on the groups of pSS patients with early or late disease onset to identify potential independent variables for a binary multivariable logistic regression model which had lymphoma as an outcome. This data driven specific algorithm has the capacity to identify, among a plethora of features/variables, those which are closely related to the outcome of interest (e.g., lymphoma) and less correlated amongst them, using the correlation coefficient as a similarity measure. The subsets of features/variables provided by the FCBF algorithm were subsequently used as potential independent variables in the binary multivariable logistic regression model for lymphoma, in young and old pSS study groups, to identify independent lymphoma associated factors for each age group (15). The implementation of the FCBF-based multivariable logistic regression approach along with the statistical analysis were performed in Python 3.6 and GraphPad 7.0a.

Based on the *post hoc* sample size and study power calculation conducted, assuming 80% study power and 95% one-sided levels of confidence, the present study sample size could detect an effect size (Odds Ratio) of 1.60 between patient groups (EpiInfo, CDC, Atlanta, Georgia, USA). In order to handle the multiple comparison testing, the original p-values were also adjusted with the Benjamini-Hochberg (B-H) procedure using 0.1 as the false discovery rate (16).

## Results

### Patients’ Characteristics

One thousand nine hundred and ninety-seven pSS patients were included in the study population. The male to female ratio in the total multicentre population was approximately 1:20 and the median age was 49 years (range: 5–88). It is noteworthy that 2 patients had disease onset at 5 years old. A female presented with severe Raynaud’s phenomenon at the age of 5 as confirmed by her parents and subsequently she developed dry mouth and dry eyes. The diagnosis was made many years later based on positive lip biopsy, anti Ro/SSA and anti La/SSB antibodies, while cryoglobulinemia with palpable purpura of lower extremities complicated the disease course. The other patient is a male who presented with recurrent SGE at the age of five as reported by his parents, accompanied by dry mouth and eyes at an older age while diagnosis was made few years later based on positive lip biopsy and positive Schirmer’s test. The number of patients from each Mediterranean country was similar: 972 (Greece) and 1,025 (Italy). Young and old pSS patients were matched with 353 (median age = 49 years, range: 44–57 years) and 285 (median age = 49 years, range: 44–54) middle aged pSS controls, respectively, according to gender and disease duration from pSS onset (**Table 1**). Although the matching process aimed to 1:1 ratio for both groups, this was not feasible since some pSS patients with early or late disease onset had a very long follow up time. These patients were also included in the study in order to avoid selection bias. Finally, 19% (n = 379) of patients were found to have disease onset ≤35 years of age, of whom 59.9% (n = 227) originated from Greece and 40.1% (n = 152) from Italy. Two hundred ninety-three (14.7%) pSS patients had disease onset ≥65 years of age, with 40.6% (n = 119) being Greeks and 59.4% (n = 174) Italians (**Table 1**). The median disease duration and age of disease onset of total young pSS patients was 12 (range: 0–68) and 29 (range: 5–35) years respectively, while for the old pSS patients the median disease duration was 5 years (range: 0–27), and the median age of pSS onset was 69 years (range: 65–88) (**Table 1**). Interestingly, the median disease duration from pSS diagnosis was 5 (range 0–34) and 3 years (range 0**-**22) for patients with early and late pSS onset group, respectively, pointing out a median timespan between pSS onset and diagnosis of 7 and 2 years, respectively. Despite the inability to match exactly in 1:1 ratio, disease duration was not statistically different between the young and old study groups and their matched middle-aged controls (p = 0.072 and p = 0.662, respectively).

**Table 1:** Demographic features of the total multicenter population.

	*pSS Onset* ≤ *35 years*				*pSS Onset* ≥ *65 years*			
	Greeks	Italians	Total	Controls	Greeks	Italians	Total	Controls
Number of patients (%)	11.4 (n = 227)	7.6 (n = 152)	19 (n = 379)	17.7 (n = 353)	6 (n = 119)	8.7 (n = 174)	14.7 (n = 293)	14.3 (n = 285)
Median age (years)	29 (5–35)	29 (10–35)	29 (5–35)	49 (44–57)	68 (65–83)	70 (65–88)	69 (n = 65–88)	49 (44–54)
Female (%)	95.5 (n = 216)	97.4 (n = 148)	96(n = 364)	94.6 (n = 334)	94.1 (n = 112)	93.7 (n = 163)	94 (n = 275)	96 (n = 273)
Median disease duration (onset-years)	12 (0–68)	11 (0–44)	12 (0–68)	11 (n = 0–45)	4 (0–27)	5 (0–22)	5 (0–27)	5 (0–27)

### Comparison of All pSS Patients With Disease Onset ≤35 (Young Study Group) or ≥65 years (Old Study Group) of Age With Middle Aged pSS Control Patients

**Figure 1:**
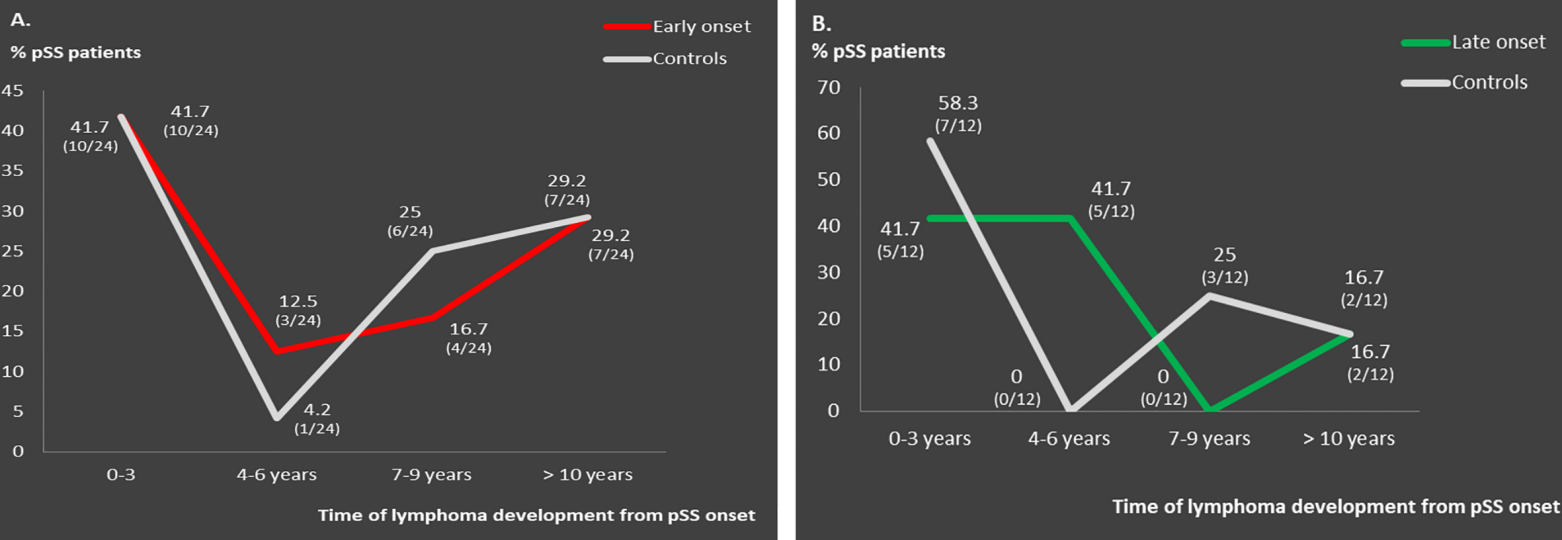
Distribution pattern of lymphoma occurrence across pSS course in: **(A)** matched early onset compared to middle-aged onset pSS lymphoma patients (n = 24) and **(B)** matched late onset compared to middle-aged onset pSS lymphoma patients (n = 12).

Clinical, laboratory and histologic features were compared between pSS patients with disease onset ≤35 or ≥65 years and their middle-aged matched pSS controls (**Tables 2** and **3**, respectively). Young pSS patients presented more frequently SGE (39.1 vs. 27.1%, p = 0.001; OR = 1.72, 95% CI: 1.25–2.35), lymphadenopathy (20.7 vs. 12.2%, p = 0.005; OR = 1.86, 95% CI: 1.21–2.83), Raynaud’s phenomenon (36.6 vs. 27.5%, p = 0.011; OR = 1.52, 95% CI: 1.11–2.10), anti-Ro/SSA (91.2 vs. 78.6%, p < 0.001; OR = 2.83, 95% CI:1.83–4.37), anti-La/SSB (47.7 vs. 34.8%, p < 0.001; OR = 1.70, 95% CI: 1.25–2.30), RF positivity (71 vs. 53.2%, p < 0.001; OR = 2.15, 95% CI: 1.57–2.95), C4 hypocomlementemia (38.4 vs. 26.2%, p = 0.001; OR = 1.75, 95% CI: 1.26–2.42), hypergammaglobulinemia (79.3 vs. 59.4%, p < 0.001; OR = 2.61, 95% CI: 1.80–3.78), leukopenia (15.4 vs. 7.4%, p < 0.001; OR = 2.36, 95% CI: 1.42–3.85), lymphoma [10.3% (n = 39/379) vs. 5.7% (n = 20/353), p = 0.030; OR = 1.91, 95% CI: 1.11–3.27], and, less often, dry mouth (86.5 vs. 94.6%, p < 0.001, OR = 2.17, 95% CI: 1.58–4.62), dry eyes (89 vs. 94.1%, p = 0.018; OR = 1.97, 95% CI: 1.14–3.45), peripheral neuropathy (1.1 vs. 3.4%, p = 0.040; OR = 3.30, 95% CI: 1.07–9.45), interstitial lung disease (ILD) (2.1 vs. 5.7%, p = 0.020; OR = 2.78, 95% CI: 1.25–6.03) and diffuse large B cell lymphomas (DLBCL) [3% (n = 1/34) vs. 26% (n = 4/15), p = 0.025; OR = 12, 95% CI: 1.56–150) compared to the middle-aged matched pSS controls (**Table 2**). No differences were identified regarding the proportion of pSS patients with FS≥1 or MALT lymphoma type [82% (n = 28/34) vs. 66% (n = 10/15), p-value = 0.317] between young patients and middle-aged controls as opposed to DLBCL [3% (1/34) vs. 26% (4/15), p-value = 0.025], although the number of cases was very small Given that SGE and lymphadenopathy can be also lymphoma manifestations, it is noteworthy that 26 and 17 of 39 young lymphoma patients had SGE and lymphadenopathy with median time prior to lymphoma diagnosis of 3 (range: 0.5–37) and 2 years (range: 0.5–17) respectively. After B-H adjustment, all the above features maintained their statistical significance with the addition of biopsy focus score of ≥1 and monoclonality, which were found more frequently in the young and control group respectively (**Table 2**). The time to lymphoma development from pSS onset between the young and middle-aged controls with lymphoma was not statistically different [median time to lymphoma development (range): 9 (0–37) vs. 8 (1–29) years, p = 0.456)]. After careful matching of 24 pSS young lymphoma patients with 24 middle aged lymphoma controls according to pSS disease duration (mean ± SE: 12.75 ± 1.435 vs. 12.88 ± 1.529, p = 0.952), the distribution of lymphoma diagnosis across the pSS course showed two incidence peaks: one peak within the first 3 years of pSS onset common for both groups (42%) and a second one after 7 years for the middle-aged controls (54%) and after 10 years for the early onset pSS patients with lymphoma (29%) (**Figure 1A**).

**Table 2:** Comparison of cumulative clinical, laboratory, and histologic features between pSS patients with early disease onset and their middle-aged pSS control patients.

Clinical and laboratory features %(n)	pSS onset≤ 35 years (n = 379)	pSS middle-aged controls (n = 353)	P value	BH-adjustment
***Sicca manifestations***				
Dry mouth Dry eyes	86.5 (327/378) 89 (336/378)	94.6 (331/350) 94.1 (332/353)	**<0.001** **0.018**	**0.011** **0.032**
***Non-specific***				
Chronic fatigue Arthralgia/myalgia Arthritis Myositis	31.7 (111/350) 62.8 (238/379) 20.9 (68/325) 1.1 (4/379)	32.7 (101/309) 65.2 (229/351) 18.6 (57/307) 2.3 (8/353)	0.854 0.541 0.519 0.249	0.094 0.079 0.073 0.061
***Vascular***				
Raynaud’s phenomenon Purpura Vasculitic ulcer	36.6 (134/366) 13.7 (52/379) 5.3 (20/379)	27.5 (95/346) 10.5 (37/353) 4.5 (16/353)	**0.011** 0.219 0.768	**0.029** 0.055 0.091
***Glandular***				
SGE Lymphadenopathy Splenomegaly Lymphoma ***PNS (vasculitic)*** ***CNS*** ***ILD***	39.1 (147/376) 20.6 (73/353) 0.5 (2/379) 10.3 (39/379) 1.1 (4/379) 1.1 (4/379) 2.1 (8/379)	27.1 (95/350) 12.2 (39/318) 0.6 (2/353) 5.7 (20/353) 3.4 (12/352) 1.7 (6/353) 5.7 (20/353)	**0.001** **0.005** 1 **0.030** **0.040** 0.533 **0.020**	**0.020** **0.026** 0.097 **0.041** **0.044** 0.076 **0.035**
***Renal***				
IRD GN	2.1 (8/379) 1.6 (6/377)	0.9 (3/353) 0.9 (3/351)	0.226 0.507	0.058 0.070
***Liver***				
* PBC* AHI Sclerosing Cholangiitis	1.3 (5/378) 1.3 (5/377) 1.3 (5/378)	2(7/353) 0.3 (1/351) 0.9 (3/351)	0.681 0.218 0.726	0.085 0.052 0.088
***Laboratory features***				
Anti-Ro Anti-La Anti-Ro/La Hypergammaglolobulinemia Low C4 Rheumatoid Factor Cryoglobulinemia Monoclonality Leukopenia Thrombocytopenia	91.2 (343/376) 47.7 (177/371) 91.7 (344/375) 79.3 (233/294) 38.4 (139/362) 71 (252/355) 11 (25/227) 5.9 (21/357) 15.4 (58/377) 3.1 (11/357)	78.6 (268/341) 34.8 (118/339) 77.9 (264/339) 59.4 (161/271) 26.2 (86/328) 53.2 (177/333) 7.9 (16/203) 9.8 (32/325) 7.1 (25/350) 2.1 (7/329)	**<0.001** **<0.001** **<0.001** **<0.001** **0.001** **<0.001** 0.347 0.073 **<0.001** 0.588	**0.008** **0.014** **0** **0.002** **0.023** **0.005** 0.067 **0.047** **0.017** 0.082
***Histologic***				
FS≥1 MALT lymphomas DLBC lymphomas	88 (155/176) 82 (28/34) 3 (1/34)	82 (136/165) 66 (10/15) 26 (4/15)	0.187 0.317 **0.025**	**0.05** 0.064 **0.038**

SGE, salivary gland enlargement; PNS, peripheral nervous system; CNS, central nervous system; ILD, interstitial lung disease; IRD, interstitial renal disease; GN, glomerulonephritis; PBC, primary biliary cirrhosis; AHI, autoimmune hepatitis; MALT, mucosa associated lymphoid tissue; DLBC, diffuse large B-cell; BH, Benjamini Hochberg, Leukopenia ≤3000/mm^3^, Thrombocytopenia ≤ 100000/mm^3^, Hypergammaglobulinemia >20 g/L.

Bold character is used to highlight those results with statistical significance.

**Table 3:** Comparison of cumulative clinical, laboratory and histologic features between pSS patients with late disease onset and their middle-aged pSS control patients.

Clinical & laboratory Features %(n)	pSS onset≥65 years (n = 293)	pSS middle-aged controls (n = 285)	P value	BH-adjustment
***Sicca manifestations***				
Dry mouth Dry eyes	96.9 (283/292) 92.8 (272/293)	92.9 (263/283) 90.9 (259/285)	**0.046** 0.479	**0.017** **0.047**
***Non-specific***				
Chronic fatigue Arthralgia/myalgia Arthritis Myositis	34.4 (83/241) 57.2 (167/292) 10.2 (24/235) 1 (3/293)	33.9 (78/230) 61.8 (176/285) 17.1 (41/240) 1.1 (3/285)	0.981 0.302 **0.040** 1	0.085 **0.038** **0.014** 0.091
***Vascular***				
Raynaud’s phenomenon Purpura Vasculitic ulcer	22.7 (66/291) 8.5 (25/293) 2.7 (8/292)	23.7 (66/278) 8.1 (23/285) 2.1 (6/284)	0.841 0.959 0.827	0.070 0.082 0.067
***Glandular***				
SGE Lymphadenopathy Splenomegaly Lymphoma ***PNS (vasculitic)*** ***CNS*** ***ILD***	23.6 (68/288) 10.8 (26/241) 0.7 (2/291) 6.8 (20/293) 5.5 (16/293) 2.1 (6/293) 7.9 (23/291)	19.4 (55/284) 13.6 (33/243) 0.4 (1/282) 2.1 (6/285) 2.1 (6/285) 1.1 (3/285) 2.5 (7/285)	0.256 0.423 1 **0.011** 0.058 0.504 **0.005**	0.032 **0.044** 0.088 **0.011** **0.020** **0.05** **0.005**
***Renal***				
IRD GN	1.7 (5/293) 1 (3/291)	1.8 (5/285) 0.4 (1/285)	0.783 0.623	0.064 0.061
***Liver***				
* PBC* AHI Sclerosing Cholangiitis	1.4 (4/293) 0.7 (2/291) 1.4 (4/291)	1.1 (3/285) 1.8 (5/284) 0.4 (1/284)	1 0.280 0.373	0.094 **0.035** **0.041**
***Laboratory features***				
Anti-Ro Anti-La Anti-Ro/La Hyperglobulinemia Low C4 Rheumatoid Factor Cryoglobulinemia Monoclonality Leukopenia Thrombocytopenia	67.9 (199/293) 30.8 (89/289) 70.2 (205/292) 43.6 (109/250) 19.6 (53/270) 47 (127/270) 10.2 (20/197) 9.9 (27/273) 11.4 (33/289) 2.8 (8/283)	79.3 (219/276) 37.5 (103/275) 80.1 (222/277) 62.1 (146/235) 17.3 (46/266) 52.4 (144/275) 5.3 (10/189) 9.1 (24/265) 10.7 (30/281) 2.2 (6/272)	**0.002** 0.114 0.008 < 0.001 0.558 0.246 0.111 0.854 0.881 0.844	**0.002** **0.026** **0.008** **0** 0.055 **0.029** **0.023** 0.076 0.079 0.073
***Histologic***				
FS ≥ 1 MALT lymphomas DLBC lymphomas	85 (107/126) 78 (14/18) 5.5 (1/18)	81.5 (110/135) 100 (6/6) 0 (0/6)	0.564 0.539 1	0.058 0.052 0.097

SGE, salivary gland enlargement; PNS, peripheral nervous system; CNS, central nervous system; ILD, interstitial lung disease; IRD, interstitial renal disease; GN, glomerulonephritis; PBC, primary biliary cirrhosis; AHI, autoimmune hepatitis; MALT, mucosa associated lymphoid tissue; DLBC, diffuse large B-cell; BH, Benjamini Hochberg, Leukopenia ≤3000/mm^3^, Thrombocytopenia ≤ 100000/mm^3^, Hypergammaglobulinemia >20 g/L

Bold character is used to highlight those results with statistical significance.

Old pSS patients had higher frequency of dry mouth (96.9 vs. 92.9%, p = 0.046; OR = 2.39, 95% CI: 1.11–5.45), ILD 7.9 vs. 2.5%, p = 0.005; OR = 3.40, 95% CI: 1.50–8.47), and lymphoma [6.8% (n = 20/293) vs. 2.1% (n = 6/285)], p = 0.011; OR = 3.40, 95% CI: 1.34-8.17) and lower frequency of arthritis (10.2 vs. 17.1%, p = 0.040; OR = 1.81, 95% CI: 1.04–3.07), anti-Ro/SSA (67.9 vs. 79.3%, p = 0.002; OR = 1.81, 95% CI: 1.23–2.68), and hypergammaglobulinemia (43.6 vs. 62.1%, p < 0.001; OR = 2.12, 95% CI: 1.48–3.06) compared to the middle aged matched controls (**Table 3**). No differences were identified regarding the percentage of pSS patients with FS ≥ 1,MALTly [78% (n = 14/18) vs. 100% (n = 6/6), p-value = 0.539) and DLBCL [5.5% (n = 1/18) vs. 0% (n = 0/6), p-value = 0.097] lymphoma type between old and middle age controls. Thirteen and 6 of 20 old lymphoma patients had SGE and lymphadenopathy prior to lymphoma diagnosis with a median time of 3 (range: 0.5–5) and 1 (0.5–2) year, respectively. After B-H adjustment, in addition to the parameters mentioned above, pSS patients with late onset displayed also more frequently dry eyes, peripheral neuropathy, involvement of the central nervous system, sclerosing cholangitis and cryoglobulinemia, and, less commonly, arthralgia/myalgia, lymphadenopathy, anti-La, anti-Ro/La, and rheumatoid factor compared to their matched middle-aged controls (**Table 3**). The time to lymphoma development from pSS onset between old and middle-aged controls with lymphoma was not statistically different [median time to lymphoma development (range): 4 (0–15) vs. 2 (1–10) years, p = 0.705)]. After careful matching of 12 pSS old lymphoma patients with 12 middle aged lymphoma controls, according to pSS disease duration (mean ± SE: 8 ± 1.59 vs. 8.33 ± 1.65, p = 0.886), the distribution of lymphoma appearance across the pSS course showed that >80% of old lymphoma pSS patients developed lymphoma within 6 years after pSS onset (**Figure 1B**).

### Comparison Between Greek and Italians pSS Patients With Disease Onset at ≤35 (Young Study Group) and ≥65 Years of Age (Old Study Group)

Clinical, laboratory and histologic features were compared between Greek and Italian pSS patients with disease onset ≤35 or ≥65 years and are presented with and without B-H adjustment in **Supplementary Table 1**. Briefly, Italian young pSS patients had significantly higher frequency of dry mouth, dry eyes, lymphadenopathy, chronic fatigue, anti-Ro/SSA positivity, and leukopenia and lower frequency of cryoglobulinemia, C4 hypocomplementemia, skin vasculitic ulcer, lymphoma, and MSG FS ≥ 1 compared to the Greek young. Italian old SS patients had higher frequency of peripheral neuropathy, chronic fatigue, anti-Ro/SSA positivity, and lower frequency of C4 hypocomplementemia, skin vasulitic ulcer, lymphoma,and MSG FS ≥ 1 compared to the Greek old group. Interestingly, leukopenia was markedly prevalent for both early and late onset pSS Italian patients compared to Greeks.

### The Effect of Age at Disease Onset on Lymphoma Development Using Data Driven Analysis

**Figure 2:**
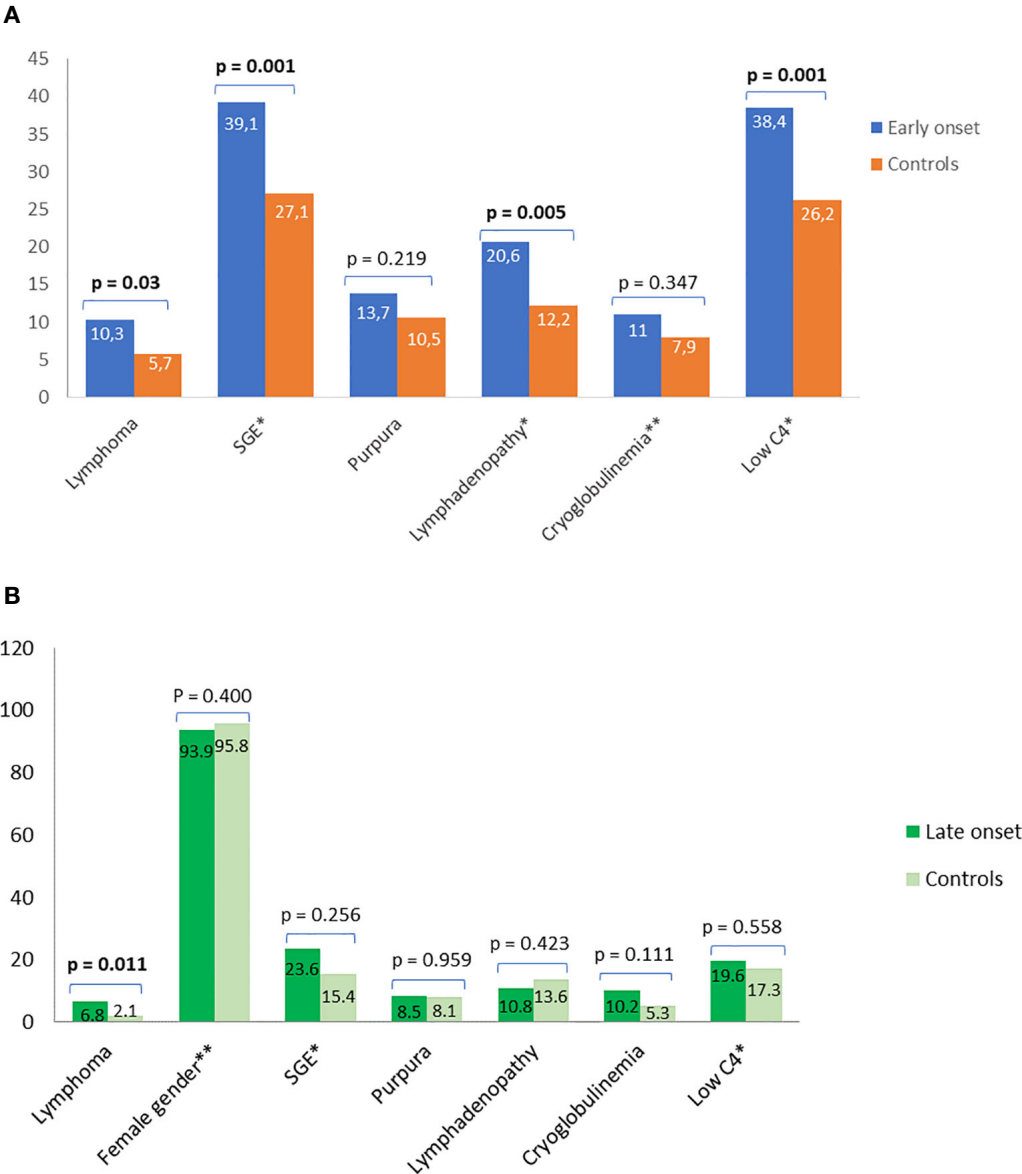
Prevalence and classical predictors for lymphoma using simple statistics and data driven approaches. Comparison of prevalence and classical predictors for lymphoma between **(A)** pSS patients with early disease onset and their matched middle-aged controls and **(B)** pSS patients with late disease onset and their matched middle-aged controls. Independent predictors for lymphoma after data driven analysis with FCBF based multivariable logistic regression analysis are shown with asterisk. The second asterisk connotes a negative association. SGE, salivary gland enlargement.

The FCBF algorithm was applied on the dataset of young pSS study group, analyzing 35 distinct features including clinical serological and laboratory data. The 6 variables in terms of magnitude of order with the strongest association with lymphoma and the weakest association among them as calculated by the FCBF algorithm were: cryoglobulinemia, low C4, lymphadenopathy, SGE, interstitial lung disease (ILD), and RF positivity, which were used to design a binary multivariable logistic regression model with lymphoma as an outcome (**Supplementary Table 2**). Cryoglobulinemia, low C4, SGE, and lymphadenopathy were identified as independent associated factors for lymphoma development among pSS patients with early disease onset (**Figure 2A**). The performance of the FCBF/LR model for the young group was good, with accuracy = 0.90, sensitivity = 0.56, and AUC = 0.84 (**Supplementary Figure 1A**). A 10-fold cross validation approach was applied to calculate the performance of the FCBF/LR model. The application of the FCBF algorithm on the dataset of the old pSS study group, analyzing the same 35 features, revealed 6 strong variables to construct a binary multivariable logistic regression model with lymphoma as an outcome: splenomegaly, SGE, low C4, female gender, dry mouth, and kidney involvement/glomerulonephritis (GN) (**Supplementary Table 3**). However, only SGE, low C4 and male gender were finally identified as independent lymphoma associated factors among pSS patients with late disease onset (**Figure 2B**). The performance of the FCBC/LR model for the old pSS study group was good with accuracy = 0.93, sensitivity = 0.52, and AUC = 0.80 (**Supplementary Figure 1B**). Similarly, the 10-fold cross validation approach was applied to calculate the performance of the FCBC/LR model.

## Discussion

This is the largest study including pSS patients with either early (≤35) or late (≥65) disease onset, stemming from a multicentre study population. The role of age at pSS onset on the clinical expression of pSS has been studied in the past but the number of included patients was limited and/or the age groups were not compared with the typical middle-aged pSS patients (3–10). To explore the effect of age on the clinical phenotype of pSS, we followed a unique study design approach: i) pSS patients fulfilling the 2016 ACR/EULAR criteria from an integrated Greek-Italian population were included in the current study, ii) pSS patients with early or late pSS onset were matched and compared with typical middle aged pSS control patients according to gender and disease duration using two distinct control groups for each age group, iii) data driven analysis was employed to identify lymphoma associated factors for each age group separately, and iv) we matched early or late onset pSS lymphoma patients with middle-aged pSS lymphoma controls to study the distribution of lymphoma occurrence during pSS course. The major findings of our study that draw clinical attention can be summarized as follows: a) young group had higher prevalence of B cell associated manifestations including SGE, hypergammaglobulinemia, presence of autoantibodies, leukopenia, C4 hypocomplementemia and lymphoma, b) old study group had more frequently dry mouth, interstitial lung disease and lymphoma, c) lymphoma associated factors were different between the 2 age groups as shown by data driven analysis, and d) lymphoma distribution across the pSS course followed different patterns depending on age of pSS onset.

The proportions of young (19%) and old (14%) pSS patients in the total population, confirm previous studies reporting a range of 9–38% for young and 6–36% for old patients, depending on the cut-offs of the age group (35–45 or 65–70 years, respectively), whether age refers to pSS onset or diagnosis and on the cohort size (3–9). Focusing on well-defined cohorts, it is anticipated that more than one third of pSS patients, have an early or late disease onset, pointing out the clinical importance to study the clinical phenotypes of these age groups. The young group is characterized by B cell hyperactivity compared to the middle-aged controls, implying more robust B cell responses in combination with classical risk factors of lymphoma such as low serum C4 levels, SGE, and leukopenia that justify the higher lymphoma prevalence. Similar findings have been described also by other groups in the past (3–8), although higher lymphoma prevalence is reported for the first time. In a previous study including only 13 pSS patients with disease onset <35 years, it was also described that this subset had increased lymphoma frequency. However, the pSS control group was not restricted only to those with disease onset at the 4^th^ or 5^th^ decade but instead included all patients with disease onset >35 (7). In line with the intense B cell responses of young patients, pSS patients of the old study group were found to have lower frequency of hypergammaglobulinemia and anti-Ro/SSA antibodies compared to the middle-aged controls, suggesting a less aggressive B cell autoimmune response with aging. In addition, the lower frequency of young pSS patients compared to middle-aged pSS controls regarding sicca manifestations, peripheral neuropathy, and interstitial lung disease may be the result of environmental factors, comorbidities, or aging itself (17). Similarly, the old group exhibit more frequently dry mouth and interstitial lung disease and less frequently arthritis, autoantibodies, and hypergammaglobulinemia. Although a proportion of old patients received minor antidepressants contributing to oral dryness, it was not statistically significant different compared to their middle aged controls. The higher prevalence of lymphoma in old pSS patients is also reported for the first time. Very few studies have been conducted to investigate the phenotype of elderly pSS patients, but none has explored this sequela (4–6, 9, 10). The slightly higher lymphoma prevalence in the apparent absence of strong B cell hyperactivity, could partially be attributed to immunosenescence, in addition to the effect of common independent lymphoma risk factors such as low C4 and SGE. An association of lymphoma prevalence and disease duration is also noteworthy. Although the middle-aged control groups differ between the young and the old pSS study groups in terms of disease duration, conclusions can be drawn regarding the physical course of the disease. The middle-aged pSS controls matched to the young group display a median disease duration of 11 years and lymphoma prevalence of 5.2%, while the middle-aged pSS controls of the old group had disease duration of 5 years with lymphoma prevalence of 2%. These data are consistent with each other and in accordance with previous studies supporting a life time risk of lymphoma in pSS between 5 and 10% (18–22), clearly underlying the quality of data and study design and the conclusion that both age groups for different reasons, are more prone to develop lymphoma.

Regarding the distribution pattern of lymphoma occurrence, young pSS lymphoma patients had one peak incidence within 3 years of pSS onset and a second peak after 10 years of disease duration. pSS patients with early onset have strong B cell responses along with classical risk factors for lymphoma. On the other hand, pSS associated lymphomas are mainly of B cell origin following a longstanding and multistep process through chronic antigenic stimulation of the B cell component that may evolve into malignant transformation by accumulation of genetic mutations in combination with failure of immunoregulatory mechanisms to control malignancy. Thus, it is possible for young pSS lymphoma patients who develop lymphoma later to present also strong immunoregulatory mechanisms that may delay the lymphomagenesis process. On the contrary, old pSS lymphoma patients present lymphoma very early during pSS course, most likely as a result of excessive immunosenescence due to aging, incapable to withhold the underlying lymphomagenesis process.

The differences in the phenotypes between Italians and Greeks, seem to share some common features in the young and the old pSS patients (e.g., chronic fatigue, anti-Ro/SSA positivity, lymphadenopathy, leukopenia, skin ulcers, low C4, and lymphoma), suggesting that genetic, environmental, and socioeconomic variations may drive the different biologic and immunologic responses between the 2 ethnic groups, independently of age. This is further supported by the fact that in both age groups, Greeks have more frequently lymphoma compared to Italians, along with traditional risk factors such as severe skin purpura with ulcer, low C4, and cryoglobulinemia.

The application of data driven analysis to build a logistic regression model for lymphoma associated factors is considered a novelty. Usually, the features/independent variables chosen by the researches to construct a logistic regression model were based on positive findings of the univariate analysis, data from the literature and/or potential biologic associations with the outcome of interest (e.g. lymphoma), underestimating the prerequisite of independency among the selected features/variable. On the contrary by using an FCBF/LR data driven approach we managed to: a) consider and analyse 35 distinct features/variables as potential independent variables for each age group b) avoid bias selection through a mathematical based algorithm c) end up with a reasonable number of potentially strong and independent variables to be managed by the logistic regression model for a given number of patients and d) reveal a subset of independent lymphoma associated factors/variables that differ between the 2 age groups and might not had been identified with the classical statistics. Data driven analysis across the total young and old groups revealed distinct combination of independent lymphoma associated factors, which can be very useful for everyday clinical practice. The prominent features/independent lymphoma associated factors in young pSS patients included traditional predictors of lymphoma that were also identified with classical statistics compared to the middle-aged controls such as SGE and C4 hypocomplementemia. However, the FCBF/logistic regression model revealed, cryoglobulinemia, and lymphadenopathy as additional age-specific lymphoma associated factors, reflecting intense B cell responses and underlining the analytic power of data driven approaches. On the contrary, the old group has different subset of prominent features/lymphoma associated factors, which did not differ compared to the middle-aged matched controls such as SGE and low C4. It is noteworthy that for both age groups, SGE and C4 hypocomplementemia are common shared features, implying that both of these factors are strongly associated with lymphomagenesis. Younger and older pSS patients with the aforementioned subsets of features/variables are considered as high risk for lymphoma development and should be closely followed up.

Inevitably, our study has some limitations. The retrospective nature of the data is definitively an important limitation that may affect the conclusions. In addition, the heterogeneity between Greek and Italian pSS patients, possibly due to genetic and environmental differences, is another limitation that may also affect data analytics, especially if other national cohorts are integrated in the analysis. Regarding the FCBF/LR model for lymphomas, there was an imbalance between lymphoma and non-lymphoma patients in both study groups, leading to relatively low sensitivity. The multiple comparison testing is also an important issue of this study. However, adjustment for multiple comparison testing is controversial, since the effort to control type I error may lead to enhanced type II error. Although B-H is considered a common adjustment procedure (16), many researchers, including us, choose not to make any adjustments (23). Therefore, we decided to present both the original and B-H adjusted p-values, respecting all scientific approaches. The fact that we have chosen the date of pSS onset as the time point to estimate disease duration and classify patients according to age is subject to recall bias and may lead to discrepancies, at least to some extent between the pSS onset and the time of data collection related to pSS. However, we feel that the onset of pSS and especially the occurrence of sicca symptoms precedes the time of pSS diagnosis, since the majority of patients seek medical advice after the establishment of clinical manifestations related to Sjogren’s syndrome. In this line, it seems that the time of pSS onset represents better the underlying pathogenetic process of the disease.

In conclusion, it is of great clinical importance to study the effect of age that may affect the clinical expression of pSS. Patients with early or late pSS onset are characterized by distinct clinical phenotypes, higher lymphoma prevalence different clusters of lymphoma associated factors, and distribution of lymphoma occurrence during the pSS course, implying different underlying pathogenetic mechanisms. The wide clinical spectrum of pSS encompasses rare subsets of pSS patients including early or late pSS onset, cryoglobulinemic, seronegative, or male patients. Thus, it is mandatory to gather a large number of pSS patients from several centers to study the phenotypic diversity of the disease. In this case, data driven analysis will provide higher quality results and prediction models for adverse outcomes of the disease such as development of lymphoproliferative disorders. Studying the diverse clinical phenotypes of the disease will allow a better clinical approach regarding diagnosis, follow up, and treatment of various subgroups of pSS patients, establishing in this way an era of precision medicine.

## Data Availability Statement

The raw data supporting the conclusions of this article will be made available by the authors, after request.

## Ethics Statement

The studies involving human participants were reviewed and approved by the local ethical committees of all involved Hospitals and Institutions, after patients’ informed consent and compliance with the General Data Protection Regulation (GDPR). Ethical approval information: University of Athens (National and Kapodistrian University of Athens Bioethics Committee) on 20/07/2017; Harokopio University (Harokopio University Bioethics Committee) on 02/03/2018; Euroclinic hospital (Euroclinic’s hospital Bioethics Committee) on 21/03/2018 ID:111; University of Ioannina (University of Ioannina Medical School bioethics committee) ID:3261:1-2-2019; University of Udine (Unique Ethical Committee of Friuli Venezia Giulia Region) approval code: CEUR-2017-Os-027-ASUIUD and protocol number: 10735, date: 19 APR 2017; and University of Pisa (Comitato Etico Regionale per la Sperimentazione Clinica della Regione Toscana) protocol number: 65394. Written informed consent to participate in this study was provided by the participants’ legal guardian/next of kin.

## Author Contributions

All authors contributed to the article and approved the submitted version.

## Funding

The project was funded by the European Union under the Horizon "H2020-EU.3.1.1. - Understanding health, wellbeing and disease – Grand Agreement: 731944.

## Conflict of Interest

The authors declare that the research was conducted in the absence of any commercial or financial relationships that could be construed as a potential conflict of interest.
