# Supplementary material for: Primary Sjögren’s Syndrome of Early and Late Onset: Distinct Clinical Phenotypes and Lymphoma Development
Source: Front Immunol. 2020 Oct 19;11:594096. doi: 10.3389/fimmu.2020.594096 (PMC7604905; doi:10.3389/fimmu.2020.594096)
Supplement: Supplementary file 1 [file Table_1.docx]

| ***Clinical & laboratory features %(n)*** | ***pSS Onset*** $\boldsymbol{\leq}$ ***35 years***    **Greeks Italians P value BH-adjustment**  **(n=227) (n=152)**  **)** | | | | ***pSS Onset ≥ 65 years***  **Greeks Italians P value BH-adjustment**  **(n=119) (n=174)** | | | |
| --- | --- | --- | --- | --- | --- | --- | --- | --- |
| ***Sicca manifestations***  Dry mouth  Dry eyes  ***Non-specific***  Chronic fatigue  Arthralgia/myalgia  Arthritis  Myositis  ***Vascular***  Raynaud’s phenomenon  Purpura  Vasculitic ulcer  ***Glandular***  SGE  Lymphadenopathy  Splenomegaly  Lymphoma  ***PNS (vasculitic)***  ***CNS***  ***ILD***  ***Renal***  IRD  GN  ***Liver***  *PBC*  AHI  Sclerosing Cholangiitis  ***Laboratory features***  Anti-Ro  Anti-La  Anti-Ro/La  Hyperglobulinemia  Low C4  Rheumatoid Factor  Cryoglobulinemia  Monoclonality  Leukopenia  Thrombocytopenia  ***Histologic***  FS≥1  MALT lymphomas  DLBC lymphomas | 82.7(187/226)  85.4(193/226)  16.8 (38/226)  63 (143/227)  22.9 (47/205)  0.4 (1/227)  37.9 (81/214)  13.2 (30/227)  8.8 (20/227)  37.1 (83/224)  11 (25/226)  0.9 (2/227)  13.6 (31/227)  0.4 (1/227)  0.4 (1/227)  2.6 (6/227)  2.6 (6/227)  1.8 (4/225)  0.9 (2/226)  0.9 (2/225)  1.8 (4/226)  87.9(197/224)  43.8 (96/219)  87.9(197/224)  81 (115/142)  52.6(113/215)  74.1(152/205)  22.7 (20/88)  4.9 (10/205)  1.3 (3/225)  1.5 (3/205)  96 (122/127)  77 (20/26)  3.8 (1/26) | 92.1 (140/152)  94.1 (143/152)  58.9 (73/124)  62.5 (95/152)  17.5 (21/120)  1.9 (3/152)  34.9 (53/152)  14.5 (22/152)  0 (0/152)  42.1 (64/152)  37.8 (48/127)  0 (0/152)  5.3 (8/152)  1.9 (3/152)  1.9 (3/152)  1.3 (2/152)  1.3 (2/152)  1.3 (2/152)  1.9 (3/152)  1.9 (3/152)  0.7 (1/152)  96.1 (146/152)  53.3 (81/152)  97.4(148/152)  77.6 (118/152)  17.7 (26/147)  66.7 (100/150)  3.6 (5/139)  7.2 (11/152)  36.2 (55/152)  5.3 (8/152)  67.3 (33/49)  100 (8/8)  0 (0/8) | **0.013**  **0.013**  **< 0.001**  0.991  0.307  0.306  0.635  0.844  **< 0.001**  0.380  **< 0.001**  0.518  **0.013**  0.306  0.306  0.483  0.483  1  0.395  0.396  0.652  **0.011**  0.091  **< 0.001**  0.572  **< 0.001**  0.156  **< 0.001**  0.478  **< 0.001**  0.060  **< 0.001**  0.2975  0.999 | **0.032**  **0.026**  **0.002**  0.091  0.055  0.052  0.082  0.088  **0.017**  0.058  **0.008**  0.076  **0.029**  **0.047**  **0.05**  0.073  0.070  0.097  0.061  0.064  0.085  **0.023**  **0.038**  **0.020**  0.079  **0.005**  **0.041**  **0.014**  0.067  **0**  **0.035**  0,011  0,044  0,094 | 95.8 (113/118)  89.9 (107/119)  17 (20/118)  56.8 (67/118)  14.4 (16/111)  0.8 (1/119)  20.5 (24/117)  5.9 (7/119)  6.7 (8/119)  20.2 (23/114)  10.2 (12/118)  1.7 (2/119)  12.6 (15/119)  1.7 (2/119)  0.8 (1/119)  8.5 (10/117)  3.4 (4/119)  0.8 (1/118)  1.7 (2/119)  0.9 (1/117)  2.6 (3/117)  59.7 (71/119)  25.2 (29/115)  62.7 (74/118)  44.7 (34/76)  34.3 (36/105)  44.4 (44/99)  16 (8/50)  6.1 (6/99)  2.5 (3/118)  0.9 (1/109)  95 (78/82)  69 (9/13)  7.6 (1/13) | 97.7 (170/174)  94.8 (165/174)  51.2 (63/123)  57.5 (100/174)  6.5 (8/124)  1.2 (2/174)  24.1 (42/174)  10.3 (18/174)  0 (0/173)  25.9 (45/174)  11.2 (14/123)  0 (0/172)  2.9 (5/174)  8 (14/174)  2.9 (5/174)  7.5 (13/174)  0.6 (1/174)  1.2 (2/173)  1.2 (2/174)  0.6 (1/174)  0.6 (1/174)  73.6 (128/174)  34.5 (60/174)  75.3 (131/174)  43.1 (75/174)  10.3 (17/165)  49.1 (83/171)  8.2 (12/147)  12.1 (21/174)  17.5 (30/171)  4 (7/174)  66 (29/44)  100 (5/5)  0 (0/5) | 0.492  0.170  **< 0.001**  0.997  0.072  1  0.561  0.258  **< 0.001**  0.332  0.923  0.166  **0.002**  **0.018**  0.406  0.910  0.620  1  1  1  0.306  **0.017**  0.123  **0.029**  0.919  **< 0.001**  0.601  0.188  0.1651  0.158  **< 0.001**  **< 0.001**  0.2778  0.999 | 0.064  0.044  0  0.082  0.026  0.094  0.067  0.05  0.011  0.058  0.079  0.041  0.014  0.020  0.061  0.073  0.035  0.091  0.088  0.097  0.055  0.017  0.029  0.023  0.076  0.002  0.070  0.047  0.038  0.008  0.032  0.005  0.052  0.085 |

***Supplementary Table 1****. Comparison of clinical, laboratory and histologic features between Greek and Italian pSS patients with early or late pSS onset.*

Abbreviations: SGE: salivary gland enlargement, PNS: peripheral nervous system, CNS: central nervous system, ILD: interstitial lung disease, IRD: interstitial renal disease, GN: glomerulonephritis, PBC: primary biliary cirrhosis, AHI: autoimmune hepatitis, MALT: mucosa associated lymphoid tissue, DLBC: diffuse large B-cell, BH: Benjamini Hochberg, Leukopenia ≤3000/mm^3^, Thrombocytopenia ≤ 100000/mm^3^, Hypergammaglobulinemia >20 g/L.

| **Supplementary Table 2.** FCBF-based multivariable logistic regression analysis for investigating the effect of early disease onset on lymphoma development*. | | | | | |
| --- | --- | --- | --- | --- | --- |
| **Prominent feature*** | **Regression coefficient** | **Odds ratio** | **p-value** | **CI upper** | **CI low** |
| **Cryoglobulinemia** | 0.727 | 2.183 | 0.008** | 6.410 | 0.746 |
| **Low C4 (< 20mg/dl)** | 0.441 | 1.214 | 0.009** | 2.479 | 0.594 |
| **Lymphadenopathy** | 0.488 | 1.344 | 0.012** | 3.013 | 0.600 |
| **SGE** | 0.328 | 0.968 | 0.04** | 2.030 | 0.462 |
| **Lung/Interstitial Disease** | 0.434 | 1.207 | 0.208 | 9.729 | 0.154 |
| **Rheumatoid Factor (RF)** | 0.174 | 0.710 | 0.152 | 1.368 | 0.368 |
| * Features/Variables analysed by the FCBC algorithm: Ethnicity, Gender, Disease duration (onset), Age at SS onset, Dry mouth, Dry eyes, Anti-Ro, Anti-La, Anti-Ro or La, RF, Monoclonal gammopathy, SGE, Lymphadenopathy, Low C4(<20mg/dl), Chronic Fatigue, Arthralgias-myalgias, Arthritis, Raynaud’s phenomenon, Palpable purpura, Vasculitic ulcer, Myositis, Peripheral Neuropathy, PNS-vasculitic, CNS involvement, Liver-sclerosing cholangitis, Liver–autoimmune hepatitis, Liver- PBC (Primary Biliary Cirrhosis), Lung – interstitial disease, Interstitial renal disease, Kidney/Glomerulonephritis, Splenomegaly, WBC≤3000/mm^3^, Thrombocytopenia ≤ 100000/mm^3^, Hypergammaglobulinemia, Cryoglobulinemia, Lymphoma | | | | | |
| ** < 0.05 (95% confidence interval) | | | | | |

| **Supplementary Table 3.** FCBF-based multivariable logistic regression analysis for investigating the effect of late disease onset on lymphoma development. | | | | | |
| --- | --- | --- | --- | --- | --- |
| **Prominent feature*** | **Regression coefficient** | **Odds ratio** | **p-value** | **CI upper** | **CI low** |
| **Splenomegaly** | 0.492 | 1.472 | 0.435 | 31.474 | 0.160 |
| **SGE** | 0.636 | 1.821 | 0.011** | 5.171 | 0.641 |
| **Low C4 (< 20mg/dl)** | 0.525 | 1.438 | 0.035** | 4.388 | 0.472 |
| **Female Gender** | -0.691 | 0.127 | 0.026** | 0.487 | 0.033 |
| **Dry Mouth** | -0.361 | 0.242 | 0.144 | 0.923 | 0.064 |
| **Kidney-GN** | -0.072 | 0.433 | 0.477 | 117.25 | 0.002 |
| * Feutures/Variables analysed by the FCBC algorithm: Ethnicity, Gender, Disease duration (onset), Age at SS onset, Dry mouth, Dry eyes, Anti-Ro, Anti-La, Anti-Ro or La, RF, Monoclonal gammopathy, SGE, Lymphadenopathy, Low C4(<20mg/dl), Chronic fatigue, Arthralgias-myalgias, Arthritis, Raynaud’s phenomenon, Palpable purpura, Vasculitic ulcer, Myositis, Peripheral Neuropathy, PNS-vasculitic, CNS involvement, Liver-sclerosing cholangitis, Liver–autoimmune hepatitis, Liver- PBC (Primary Biliary Cirrhosis), Lung – interstitial disease, Interstitial renal disease, Kidney/Glomerulonephritis, Splenomegaly, WBC≤3000/mm^3^, Thrombocytopenia ≤ 100000/mm^3^, Hypergammaglobulinemia, Cryoglobulinemia, Lymphoma | | | | | |
| ** < 0.05 (95% confidence interval) | | | | | |


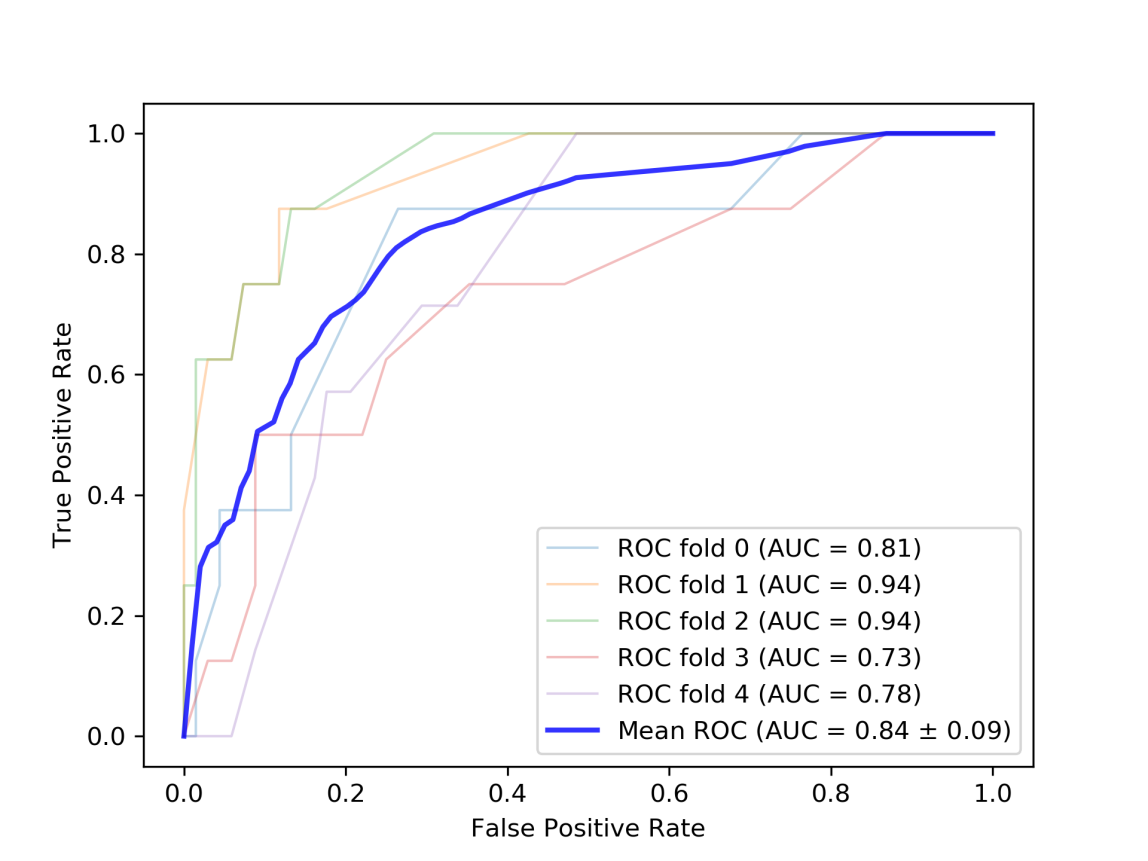


**
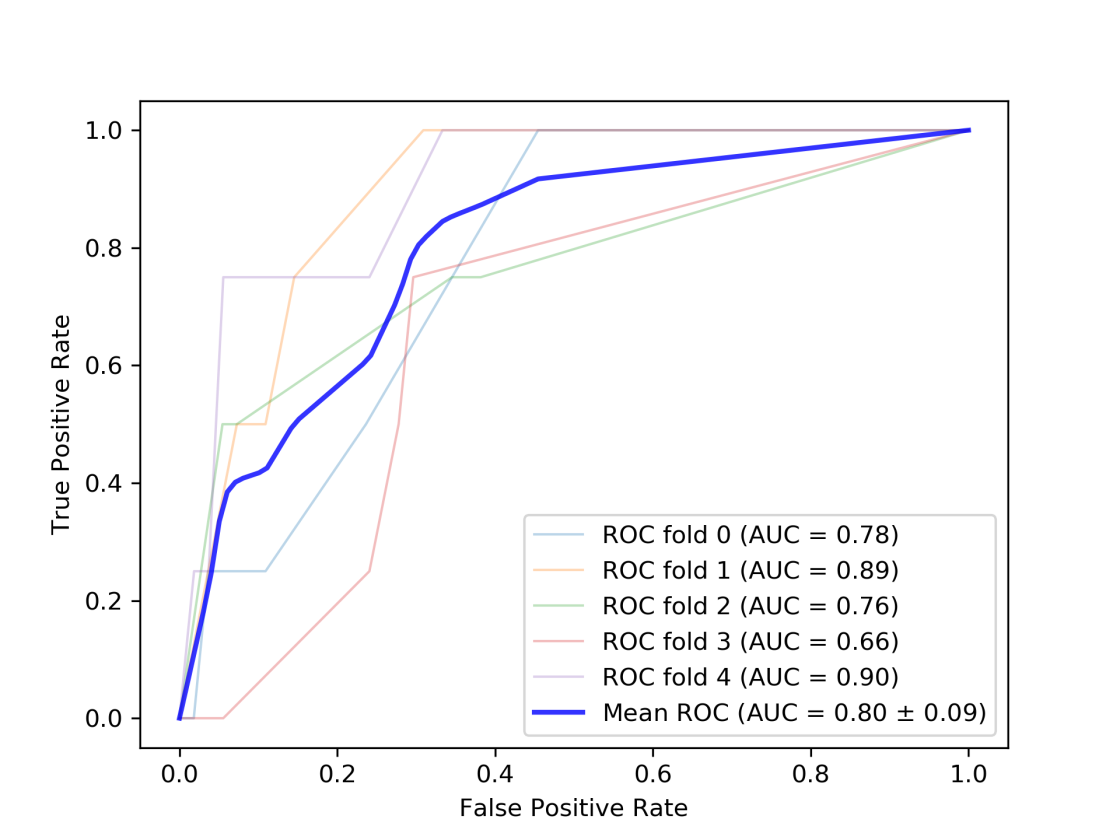
**

**Supplementary Figure 1**.

Performance and ROC curves of FCBF based multivariable logistic regression prediction model for lymphoma prediction in A. SS patients with early disease onset and B. SS patients with late disease onset.

Ethnicity, Gender, Disease duration (onset), Age at SS onset, Dry mouth, Dry eyes, Anti-Ro, Anti-La, Anti-Ro or La, RF, Monoclonal gammopathy, SGE, Lymphadenopathy, Low C4(<20mg/dl), Chronic fatigue, Arthralgias-myalgias, Arthritis, Raynaud’s, Palpable purpura, Vasculitic ulcer, Myositis, Peripheral Neuropathy PNS-vasculitic CNS involvement, Liver-sclerosing cholangitis, Liver–autoimmune hepatitis, Liver- PBC, Lung – interstitial disease, Interstitial renal disease, Kidney/Glomerulonephritis, Splenomegaly, WBC≤3000/mm^3^, Thrombocytopenia ≤ 100000/mm^3^, Hypergammaglobulinemia, Cryoglobulinemia, Lymphoma
